# Multi-Target and Multi-Session Transcranial Direct Current Stimulation in Patients With Prolonged Disorders of Consciousness: A Controlled Study

**DOI:** 10.3389/fnins.2021.641951

**Published:** 2021-09-08

**Authors:** Xu Zhang, Baohu Liu, Yuanyuan Li, Guoping Duan, Jun Hou, Dongyu Wu

**Affiliations:** Department of Rehabilitation, Wangjing Hospital, China Academy of Chinese Medical Sciences, Beijing, China

**Keywords:** disorders of consciousness, non-invasive brain stimulation, transcranial direct current stimulation, brain networks, non-linear dynamics, electroencephalography

## Abstract

**Objectives:** To investigate the effect of multi-session transcranial direct current stimulation (tDCS) over the prefrontal area, left dorsolateral prefrontal cortex (DLPFC), and bilateral fronto-temporo-parietal cortices (FTPCs) in patients with prolonged disorders of consciousness (DOC) and to examine the altered cortical interconnections using non-linear electroencephalography (EEG).

**Methods:** In this open-label controlled study, conventional treatments were implemented in both the control and tDCS groups, together with 80 tDCS sessions only in the tDCS group. The order of tDCS targets was as follows: prefrontal area, left FTPC, right FTPC, and left DLPFC. The Coma Recovery Scale-Revised (CRS-R) and non-linear EEG index were evaluated before and after the treatment. Additionally, the modified Glasgow Outcome Scale (mGOS) was used as a follow-up evaluation at 12 months after the disease onset.

**Results:** The CRS-R improved significantly in both groups after the treatment. However, the CRS-R and mGOS were more significantly improved in the tDCS group than in the control group. Among the cross approximate entropy (C-ApEn) indices, the local C_A_-P_A_ and C_A_-F_A_ under the affected painful stimulus condition and all local and remote indices of the unaffected side under the unaffected painful stimulus condition were significantly higher in the tDCS group than in the control group. Multivariate logistic regression analysis revealed that group and type were the main relevant factors based on mGOS improvement. Multivariate linear regression analysis revealed that group, C_A_-F_A_, and C_U_-MT_U_ were the main relevant factors based on CRS-R improvement under the affected painful stimulus conditions, whereas only C_U_-MT_U_ and C_U_-FP_U_ were relevant under the unaffected painful stimulus condition.

**Conclusion:** Multi-target and multi-session tDCS could improve the cortical connections between the primary sensorimotor and frontal cortices of the affected hemisphere and the prefrontal-parietal and temporo-parietal associative cortical networks of the unaffected hemisphere. Thus, this tDCS protocol may be used as an add-on treatment for prolonged DOC.

## Introduction

Disorders of consciousness (DOC), which include conditions such as coma, vegetative state/unresponsiveness wakefulness syndrome (VS/UWS), minimally conscious state (MCS), and emerging from MCS, are characterized by varying levels of decrease in consciousness that can last from days to years or permanently (Pisa et al., 2014). Most of the survivors who wake up from coma after severe traumatic brain injury (TBI) and cerebral hemorrhage still have to face prolonged DOC (Thibaut et al., 2019). However, the management of patients with prolonged DOC remains a challenge, particularly with respect to their therapeutic options. Although some recommendations have been provided as guidelines (Giacino et al., 2018), an established and effective evidence-based medical treatment plan is currently unavailable. Fortunately, developments in the novel field of non-invasive brain stimulation brings great hope to the families of these patients with prolonged DOC.

Non-invasive brain stimulation techniques, such as transcranial direct current stimulation (tDCS), repeated transcranial magnetic stimulation, transcutaneous auricular vagal nerve stimulation, and low-intensity focused ultrasound pulse, have been used in both, the clinical and research settings. However, Thibaut et al. (2019) reviewed 14 randomized controlled trials (RCTs) and found that only 10 of these investigated the effects of non-invasive brain stimulation and indicated that only tDCS showed a clinical effect, especially in patients with MCS. Typical clinical studies on the treatment of DOC with tDCS (Angelakis et al., 2014; Thibaut et al., 2014, 2017; Naro et al., 2015; Bai et al., 2017; Dimitri et al., 2017; Estraneo et al., 2017; Huang et al., 2017; Martens et al., 2018, 2019; Cavinato et al., 2019; Guo et al., 2019; Wu et al., 2019) are listed in Table 1. Most of these studies showed that tDCS seemed effective in patients with DOC, even though the target area and therapeutic parameters were different. However, some studies that used the same design and treatment scheme arrived at contradictory conclusions (Estraneo et al., 2017; Thibaut et al., 2017).

**TABLE 1:** Overview of the studies on transcranial direct current stimulation (tDCS) for disorders of consciousness (DOC).

Authors, year	Type of study	Patient	Target	Sessions	Evaluation	Results	Follow-up
Thibaut et al., 2014	Crossover	25 UWS, 30 MCS	DLPFC	1	CRS-R	Transiently positive in MCS	12 months
Angelakis et al., 2014	Case series	7 UWS, 3 MCS	Left S1M1 or DLPFC	5	CRS-R	Suspected positive	12 months
Naro et al., 2015	Crossover	20 HC, 10 UWS, 12 MCS, 2 EMCS, 1 LIS	OFC	1	CRS-R	Positive	–
Bai et al., 2017	Crossover	9 UWS, 7 MCS	DLPFC	1	TMS-EEG	Positive	–
Thibaut et al., 2017	Crossover	16 MCS	DLPFC	5	CRS-R	Positive in some MCS	1 week
Estraneo et al., 2017	Crossover	7 UWS, 6 MCS	DLPFC	5	CRS-R; EEG	Negative	3 months
Dimitri et al., 2017	Single-case	1 MCS	DLPFC and cerebellar	36	DOCS	Positive	–
Huang et al., 2017	Crossover	37 MCS	PPC	5	CRS-R	Positive; follow-up: Negative	5 days
Martens et al., 2018	Crossover	27 MCS	DLPFC	20	CRS-R	Positive	8 weeks
Cavinato et al., 2019	Crossover	12 UWS, 12 MCS	DLPFC	10	CRS-R; WNSSP; EEG	Positive in MCS	–
Guo et al., 2019	Case series	5 UWS, 6 MCS	Precuneus	28	CRS-R; EEG	Positive	–
Wu et al., 2019	RCT	8 UWS, 7 MCS	DLPFC	10	CRS-R	Positive	3 months
Martens et al., 2019	Crossover	4 UWS, 6 MCS	Prefrontal	1	CRS-R	Negative	–

*RCT, randomized controlled trial; CRS-R, Coma Recovery Scale-Revised; DLPFC, dorsolateral prefrontal cortex; SO, supraorbital region; S1M1, primary sensorimotor cortex; OFC, orbitofrontal cortex; PPC, posterior parietal cortex; UWS, unresponsive wakefulness syndrome; MCS, minimally conscious state; EMCS, emergence of the minimally conscious state; HC, healthy individuals; LIS, locked-in syndrome; EEG, electroencephalograthy; DOCS, disorders of consciousness scale; WNSSP, Western Neurosensory Stimulation Profile; Positive, tDCS improved behavioral responses in DOC patients; Negative, tDCS did not improve behavioral responses in DOC patients.*

The choice of the target area lies at the core of the mechanism of action of tDCS in DOC. The brain networks of consciousness regulation are complex, and arousal and awareness have been defined as two linearly correlated components of consciousness (Zeman, 2001). While the bilateral fronto-temporo-parietal cortices (FTPCs) are known to participate in mediating “external awareness,” the midline anterior cingulate/medial prefrontal cortex and posterior cingulate cortices/precuneus are known to be involved in “internal awareness” (Vanhaudenhuyse et al., 2011). Recently, many studies have shown the role of strengthening fronto-parietal network connectivity in the treatment of DOC (Bai et al., 2018; Cavinato et al., 2019; Hermann et al., 2020). However, only a limited number of target areas were evaluated in most studies on tDCS for the treatment of DOC. As shown in Table 1, the left dorsolateral prefrontal cortex (DLPFC), primary sensorimotor cortex (M1), orbitofrontal cortex (OFC), and posterior parietal cortex/precuneus were chosen as targets and yielded positive tDCS results in DOC. Among these targets, the left DLPFC was the top choice. Some studies have also shown that single-target tDCS can affect the local or remote networks (Yoon et al., 2016; O’Neil-Pirozzi et al., 2017), which are based on the relatively intact remote neural network of patients. Obviously, the neural network injuries in patient with prolonged DOC are more serious. Therefore, whether single-target stimulation is enough to wake up the whole network of consciousness is elusive. Moreover, whether multi-target stimulation would be more beneficial to the recovery of DOC is obscure.

Training interventions usually produce poor effects unless they are both long-term and intense (Caeyenberghs et al., 2018). With respect to tDCS, the neuromodulation effects can be long-lasting or not depending on the session of stimulation and current intensity (Zhao et al., 2017). From Table 1, it is clear that studies using a limited number of sessions of tDCS could yield either positive or negative results. Even though studies that used more than 10 sessions of tDCS yielded positive results, these results were only observed in cases of MCS and not in those of UWS. Moreover, the positive results were more frequent in patients undergoing electrophysiological or neuroimaging assessment rather than behavioral assessment. From a clinical perspective, it is quite difficult for conventional treatments (i.e., multimodal sensory and auditory stimulation or bedside conventional physical therapy) to produce an improvement in patients with prolonged DOC, because neural plasticity and functional reorganization are time-consuming processes. Thus, producing behavioral changes in patients with prolonged DOC by using a single session or limited sessions of tDCS is nearly impossible. This highlights the need for multi-session treatment which has gained popularity in recent years, as shown in Table 1.

At present, most of the clinical research on tDCS use in DOC is limited to case reports or crossover studies (Table 1). Moreover, most of these studies were small sample trials in which the duration and severity of brain injury were obviously different. These factors will affect the reliability and validity of the research conclusions. Therefore, no recommendations regarding practice guidelines could be made based on these studies (Lefaucheur et al., 2017). Unfortunately, it is also difficult to design RCTs involving multi-target and multi-session tDCS in patients with prolonged DOC. Firstly, completing the entire treatment cycle will take a long time (several months). Secondly, one of the responses to tDCS is an increase in muscle tone (Nitsche and Paulus, 2000), which makes it difficult to blind the assessors to the treatment effects. Thirdly, patients with prolonged DOC require maximum curative effects, and this poses an ethical concern when designing an RCT. Therefore, an RCT or crossover design was not applied in this study. Instead, a historical control study design was used to compare the effects of multi-session tDCS to those of conventional treatment on prolonged DOC.

Additionally, we have noticed functional regulation of brain networks in our previous studies. Although positive results could be obtained with a single-target and limited-session tDCS in patients with aphasia (Wang et al., 2013, 2019; Wu et al., 2015), dysphagia (Yuan et al., 2017, 2019), or muscle tone issues (Wu et al., 2013), more often than not, multi-target and multi-session tDCS has been used in patients with severe brain injury. Forty-session tDCS over the prefrontal area and left DLPFC was used to improve the prognosis of patients with psychomotor inhibition state, in whom the electrophysiological results showed increased excitability in the local and remote cortical networks, especially from the sensorimotor cortex to the prefrontal cortex (Zhang et al., 2020).

In this study, according to the neural regulation mechanism of the consciousness network and our pilot study, four tDCS target areas were chosen, namely, the prefrontal area, left DLPFC, and bilateral FTPCs. Multi-session tDCS (80 sessions) was also used. The Coma Recovery Scale-Revised (CRS-R) was applied as a behavioral assessment, and the modified Glasgow Outcome Scale (mGOS) was used as a follow-up evaluation at 12 months after the onset. All patients underwent non-linear electroencephalographic (EEG) assessment to obtain information about the interconnections of the residual cortical functional islands with the associative cortices.

## Materials and Methods

### Patients

This study was conducted at the Department of Rehabilitation, Xuanwu Hospital of Capital Medical University, and Wangjing Hospital of China Academy of Chinese Medicine Sciences. The cohort of consecutive inpatients with DOC (57 with UWS and 48 with MCS) included 59 with cerebral hemorrhages and 46 with severe TBIs. This cohort enrolled patients from 2009 to 2019 which included 77 males and 28 females aged 16–83 years. The duration of disease ranged from 60 to 298 days (median, 115 days). All patients were right-handed, as identified using the Edinburgh Handedness Inventory by inquiring their spouses or guardians. Among the 105 patients, 50 recruited from 2009 to 2014 did not receive tDCS treatment and served as the control group, while the remaining 55 patients recruited from 2015 to 2019 received multi-target and multi-session tDCS and served as the experimental group. The ethics committees of both the hospitals approved this study. Written informed consents were also obtained from their spouses or guardians.

The inclusion criteria were as follows: (1) all patients had TBI or cerebral hemorrhage; (2) the diagnosis of MCS or UWS was confirmed according to the clinical definitions of the Multi-Society Task Force Report on VS (Ashwal and Cranford, 1995) and those of Giacino on MCS (Giacino et al., 2002); (3) the onset of DOC was from 2 to 10 months ago; and (4) the patients had no previous brain injury history.

The exclusion criteria were as follows: (1) unstable vital signs, (2) severe spasticity, (3) obvious communicating or obstructive hydrocephalus, (4) epilepsy, and (5) regional skin injury under the tDCS electrode.

### Design and Procedures

**FIGURE 1:**
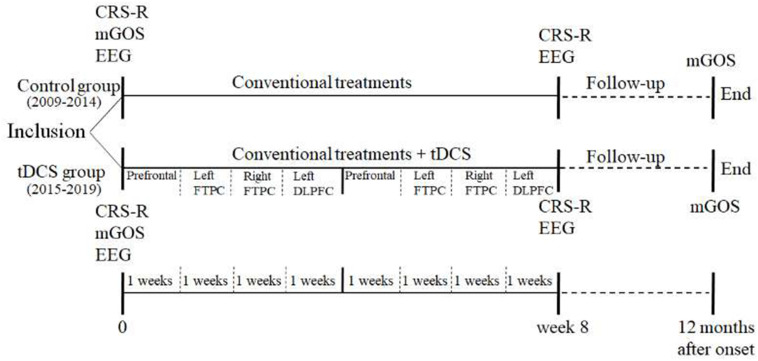
Study protocols.

This was an open-label controlled study. The patients enrolled from 2009 to 2014 did not receive tDCS treatment and served as the control group (historical control), while those recruited from 2015 to 2019 received two cycles of tDCS (80 sessions) and served as the experimental group (tDCS group). Both groups received identical conventional treatments. The order of tDCS targets was as follows: prefrontal area, left FTPC, right FTPC, and left DLPFC. Each target was treated 2 times/day for 5 days/week, and it took 4 weeks in treating all four targets (40 sessions/cycle). Each patient underwent two cycles of tDCS (total 80 sessions). The CRS-R and non-linear EEG index were evaluated before and after the treatment. Additionally, the mGOS was used to evaluate the prognosis of DOC at 12 months after the onset. Figure 1 shows the flowchart of this study.

### Interventions

A portable battery-driven device (IS200, Chengdu, China) was used to deliver a constant current of 2.0 mA (0.056 mA/cm^2^) for 20 min to the prefrontal area and bilateral FTPCs by using a pair of saline-soaked surface sponge electrodes (5 cm × 7 cm), while a current of 1.2 mA (0.056 mA/cm^2^) was applied using 4.3 cm × 5 cm electrodes to the left DLPFC.

**FIGURE 2:**
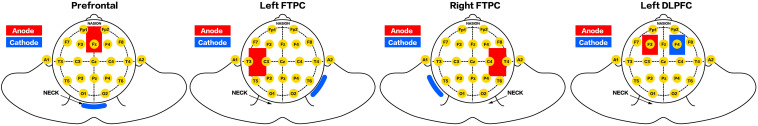
Localization of the transcranial direct current stimulation (tDCS) electrodes.

According to the results of our preliminary study and the international 10–20 system, the prefrontal area was located at 3.5 cm above the FPz (the electrode was placed vertically with its lower edge at the FPz level); the left DLPFC was identified at F3 (O’Neil-Pirozzi et al., 2017); and the left and right FTPCs were identified at the midpoints of C3 and T3 and C4 and T4, respectively. The cathode electrode was placed over the neck (prefrontal area), F4 (left DLPFC), and the back of the opposite shoulder (bilateral FTPCs) (Figure 2).

The conventional treatments, including multimodal sensory and auditory stimulations, bedside conventional physical therapy, and environmental enrichment therapy, were applied twice daily for 50 min.

### Clinical Assessment

The CRS-R (Kalmar and Giacino, 2005), which yielded a total score of 23, was used as the primary behavioral indicator to assess the patients’ consciousness by accounting for their motor function, visual function, auditory function, communication, oromotor/verbal function, and arousal. A patient’s diagnosis was based on the highest score obtained at least five successive CRS-R assessments during 1 week. The mGOS had six designations: death, VS, MCS, severe disability, moderate disability, and good recovery (Li et al., 2015).

### Electroencephalography Recording

The EEG signals (bandwidth, 0.3–100 Hz; sample rate, 500 Hz) were recorded using a wireless digital EEG system (ZN16E, Chengdu, China) under the eyes-closed and painful stimulus conditions. To reach the maximal effect of cortical activation, the acupoints LI4, ST36, LI11, SP6, SJ5, KI1, LR3, and PC6 were electrically stimulated as painful stimuli (the affected side first, and then the unaffected side) by using a Han’s acupoint nerve stimulator while EEG was being performed. The operation process of EEG recording and the chosen method of artifact-free epoch were the same as that described in previous studies (Wu et al., 2011a; Zhang et al., 2020).

In this study, 16 EEG electrodes was used according to the international 10–20 system. Impedance of electrode/skin conductance was carefully kept less than 5 kΩ for each electrode. The reference were earlobe electrodes. The description of the affected or unaffected side was used instead of the traditional single or even number accordingly because the affected sides of patients with brain injury were variable. Thus, the subscripts of the 16 electrodes were changed to: FP_U_, FP_A_, F_U_, F_A_, AT_U_ (anterior temporal), AT_A_, C_U_, C_A_, MT_U_ (middle temporal), and MT_A_, P_U_, P_A_, PT_U_ (posterior temporal), PT_A_, O_U_, and O_A_.

### Non-linear Index: Cross Approximate Entropy

Cross approximate entropy (C-ApEn) was used to analyse two related time series and measure their degree of asynchrony by comparing sequences from one series to those of the second series to reflect the spatial decorrelation of cortical potentials from two remote sites (Richman and Moorman, 2000). Higher values of C-ApEn indicated higher degrees of inter-cortical communication or information flow (Wu et al., 2011b).

The expression formula was as follows:

(1) $$\text{Cross}-\mathrm{ApEn}\left(m,r,N\right)\left(v∥u\right)=\varphi^{\text{m}}\left(r\right)\left(v∥u\right)-\varphi^{m+1}\left(r\right)\left(v∥u\right)$$

The specific parameters and details of the formula are similar to those in a previous study (Wu et al., 2011a).

The local C-ApEn (i.e., C_U_-F_U_, C_U_-MT_U_, C_U_-P_U_, C_A_-F_A_, C_A_-MT_A_, and C_A_-P_A_) and remote C-ApEn (i.e., C_U_-FP_U_, C_U_-O_U_, C_A_-FP_A_, and C_A_-O_A_) were calculated to illustrate the correlation between the changes in C-ApEn and the impaired transmission of information over short or long distances.

### Statistical Analysis

IBM SPSS Statistics for Windows, Version 22.0 (IBM Corp., Armonk, NY, United States) was used for statistical analyses. The *t*-test was used to test the difference between the two groups, and Mann–Whitney U test was used to test if the data were normally distributed. Wilcoxon signed-rank test was used for comparisons before and after the treatment. The chi-square test was used for analysing countable data. A multiple linear regression model was established using backward selection. The improvement in mGOS was regarded as the dependent variable for logistic regression. The baseline indices were included in the model for univariate and multivariate logistic regression analyses. Variables with *p* < 0.1 in the univariate logistic regression analysis were included in the multivariate logistic regression analysis. The effects of baseline data and C-ApEn on the improvement in CRS-R were investigated using univariate and multivariate linear regression analyses. A value of *p* < 0.05 was considered statistically significant.

## Results

The clinical characteristics of the patients are listed in Table 2. No significant differences in age, sex, lesion, type, duration, CRS-R, and mGOS were observed between the two groups. None of the patients withdrew from the study, and none–even those undergoing decompressive craniotomy or cranioplasty–experienced any significant adverse events. Only local flushing under the tDCS electrodes was observed in several patients.

**TABLE 2:** Clinical characteristics of the patients.

	Total (*N* = 105)	tDCS group (*N* = 55)	Control group (*N* = 50)	*P*
Age, year	48.19 ± 15.1	50.55 ± 15.84	45.6 ± 13.94	0.094
Sex				0.303
Male	77 (73.3)	38 (69.1)	39 (78)	
Female	28 (26.7)	17 (30.9)	11 (22)	
Lesion				0.253
Trauma	46 (43.8)	27 (49.1)	19 (38)	
Hemorrhage	59 (56.2)	28 (50.9)	31 (62)	
Type				0.737
UWS	57 (54.3)	29 (52.7)	28 (56)	
MCS	48 (45.7)	26 (47.3)	22 (44)	
Duration, day	115 (60,298)	101 (60,298)	124 (60,290)	0.143
Pre-CRS-R	10 (4,19)	10 (5,19)	10 (4,18)	0.800
Pre-MGOS	2 (2,3)	2 (2,3)	2 (2,3)	0.738

*Values are mean ± SD, number (percentage) or median (range).*

### Clinical Assessment

**FIGURE 3:**
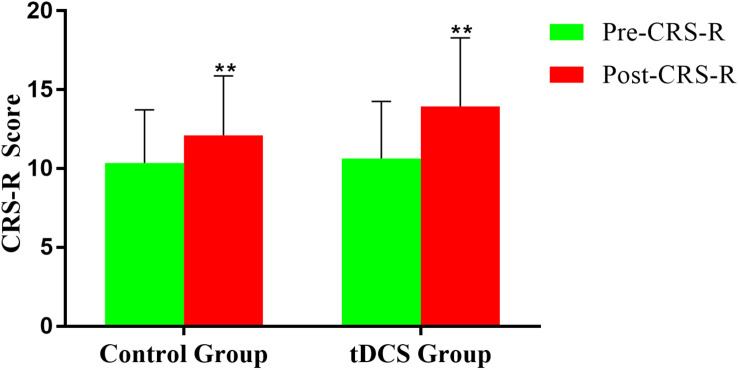
Coma Recovery Scale-Revised (CRS-R) improvement before and after treatment in the groups. **p < 0.01.

**FIGURE 4:**
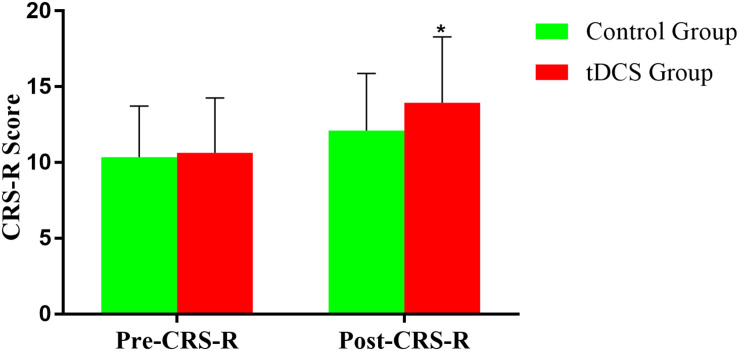
Comparison of CRS-R improvement between the two groups before and after treatment. *p < 0.05.

Coma Recovery Scale-Revised improved significantly in both groups after the treatment (Figure 3 and Tables 2, 3). The CRS-R was improved from 10 (5,19) to 16 (7,21) after the treatment in tDCS group; and the CRS-R was improved from 10 (4,18) to 13 (4,19) after the treatment in control group. There is no significant differences between both groups in CRS-R (*p* < 0.001) and mGOS (*p* = 0.016) before the treatment, but the tDCS group showed more significant improvements in CRS-R and mGOS after active tDCS treatment compared to the control group (Figure 4 and Table 3).

**TABLE 3:** Coma Recovery Scale-Revised (CRS-R) and modified Glasgow Outcome Scale (mGOS) score outcomes.

	Total (*N* = 105)	tDCS group (*N* = 55)	Control group (*N* = 50)	*P*
CRS-R				
Post-CRS-R	14 (4,21)	16 (7,21)	13 (4,19)	**0.012**
CRS-R improvement	2 (0,12)	3 (0,12)	1 (0,6)	**<0.001**
MGOS				
Follow-up-mGOS	3 (1,6)	4 (2,6)	3 (1,5)	**0.033**
mGOS improvement	1 (−1,3)	1 (0,3)	0 (−1,2)	**0.010**
mGOS improvement				**0.016**
no	50 (47.6)	21 (38.2)	29 (58)	
yes	55 (52.4)	34 (61.8)	21 (42)	

*Values are number (percentage) or median (range). Bold significant p-value: p < 0.05 was considered statistically significant.*

### Non-linear EEG Analysis

**FIGURE 5:**
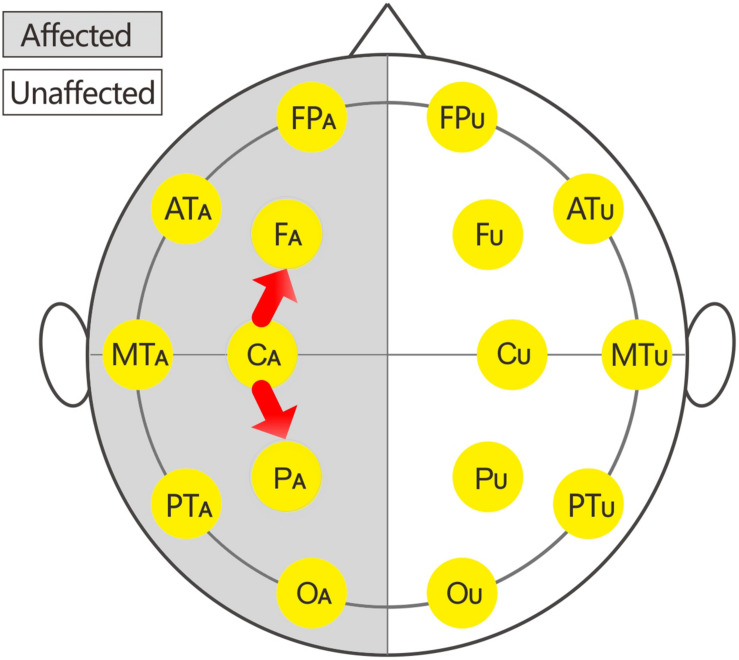
Changes in the cross approximate entropy (C-ApEn) indices under the affected painful stimulus conditions.

**FIGURE 6:**
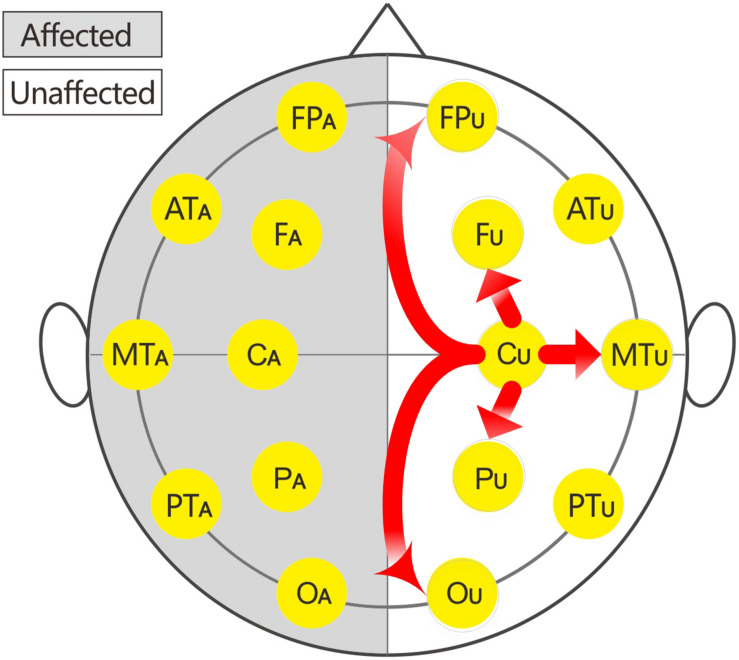
Changes in the C-ApEn indices under the unaffected painful stimulus conditions.

The EEG C-ApEn differences between the painful stimulus and eyes-closed conditions before and after the treatments are listed in Table 4. Compared to the control group, the tDCS group showed significantly higher local C-ApEn indices of C_A_-P_A_ and C_A_-F_A_ under the affected painful stimulus condition (Figure 5) and significantly higher local and remote C-ApEn indices of the unaffected side under the unaffected painful stimulus condition (Figure 6).

**TABLE 4:** Changes in the cross approximate entropy (C-ApEn) difference between the painful stimulus conditions and the eyes-closed condition before and after the treatments.

	Under the affected painful stimulus conditions			Under the unaffected painful stimulus conditions		
	tDCS group (*N* = 55)	Control group (*N* = 50)	*P*	tDCS group (*N* = 55)	Control group (*N* = 50)	*P*
Ca-Fa	0.03 (−0.17,0.3)	0.00 (−0.09,0.14)	**0.026**	0.02 (−0.25,0.2)	0.00 (−0.15,0.11)	0.466
Ca-Pa	0.04 (−0.2,0.35)	0.02 (−0.18,0.19)	**0.002**	0.02 (−0.31,0.22)	0.02 (−0.12,0.16)	0.805
Ca-MTa	0.03 (−0.22,0.22)	0.01 (−0.1,0.14)	0.183	0.02 (−0.19,0.27)	0.03 (−0.16,0.17)	0.805
Ca-FPa	0.02 (−0.14,0.33)	0.01 (−0.13,0.23)	0.062	0.02 (−0.23,0.22)	0.02 (−0.11,0.15)	0.697
Ca-Oa	0.03 (−0.25,0.23)	0.01 (−0.12,0.17)	0.251	0.01 (−0.26,0.22)	0.01 (−0.1,0.14)	0.381
Cu-Fu	0.02 (−0.14,0.18)	0.03 (−0.1,0.14)	0.319	0.07 (−0.07,0.33)	0.02 (−0.11,0.15)	**<0.001**
Cu-Pu	0.03 (−0.18,0.35)	0.02 (−0.11,0.19)	0.933	0.07 (−0.12,0.27)	0.01 (−0.12,0.15)	**<0.001**
Cu-MTu	0.04 (−0.22,0.28)	0.01 (−0.13,0.14)	0.244	0.09 (−0.04,0.43)	0.01 (−0.11,0.16)	**<0.001**
Cu-FPu	0.02 (−0.16,0.28)	0.02 (−0.15,0.20)	0.069	0.08 (−0.08,0.36)	0.00 (−0.13,0.13)	**<0.001**
Cu-Ou	0.03 (−0.17,0.3)	0.00 (−0.09,0.14)	0.495	0.07 (−0.06,0.24)	0.01 (−0.09,0.11)	**<0.001**

*Values are median (range). Bold significant p-value: p < 0.05 was considered statistically significant.*

### Regression Analysis

Table 5 lists the results of the univariate and multivariate logistic regression analyses based on mGOS improvement (≥1). These results showed that group and type were the main relevant factors. Table 6 lists the results of the multivariate linear regression analysis based on CRS-R improvement under the affected painful stimulus conditions. This analysis showed that group, C_A_-F_A_, and C_U_-MT_U_ were the main relevant factors of CRS-R improvement. However, C_U_-MT_U_ and C_U_-FP_U_ were the main relevant factors of CRS-R improvement under the unaffected painful stimulus conditions (Table 7).

**TABLE 5:** Logistic regression analysis based on mGOS improvement.

Characteristics (ref)	Univariate			Multivariate		
	OR	95%CI	*P*	OR	95%CI	*P*
Age	1.01	0.99, 1.04	0.310			
Sex (female)	1.38	0.58, 3.3	0.462			
Duration	1.00	0.99, 1	0.664			
Lesion (hemorrhage)	2.17	0.98, 4.77	**0.055**			
Type (MCS)	0.20	0.09, 0.46	**<0.001**	0.19	0.08, 0.45	**<0.001**
Group (control)	2.24	1.02, 4.89	**0.044**	2.42	1.03, 5.68	**0.042**

*Bold significant p-value: p < 0.1 in the univariate regression analysis were included in the multivariate regression analysis.*

**TABLE 6:** The relevant factors for CRS-R improvement based on multivariate linear regression analysis (under the affected painful stimulus condition).

Characteristics (ref)	Unstandardized coefficient		Standardized coefficient	*t*	*p*
			
	*B*	Standard error	*b*		
Group (control)	1.278	0.450	0.263	2.840	**0.005**
C-ApEn improvement					
Ca-Fa	7.045	3.332	0.196	2.114	**0.037**
Cu-MTu	4.942	2.692	0.168	1.836	**0.069**
Constant	1.578	0.323		4.881	<0.001

*Bold significant p-value: p < 0.1 in the univariate regression analysis were included in the multivariate regression analysis.*

**TABLE 7:** The relevant factors for CRS-R improvement based on multivariate linear regression analysis (under the unaffected painful stimulus condition).

Characteristics (ref)	Unstandardized coefficient		Standardized coefficient	*T*	*p*
			
	*B*	Standard error	*b*		
C-ApEn improvement					
Cu-MTu	8.351	3.223	0.282	2.591	**0.011**
Cu-FPu	5.638	3.370	0.182	1.673	**0.097**
Constant	1.821	0.273	<0.001	6.679	<0.001

*Bold significant p-value: p < 0.1 in the univariate regression analysis were included in the multivariate regression analysis.*

## Discussion

To date, this study is the first to present a controlled protocol of multi-target and multi-session tDCS for patients with prolonged DOC. The results confirmed that multi-session tDCS over the prefrontal area, left DLPFC, and bilateral FTPCs could improve the prognosis of patients with prolonged DOC. Compared to the control group, the tDCS group showed a significantly improved behavioral response and prognosis. Moreover, after active tDCS, the C-ApEn indices were significantly higher in the local cortical network (C_A_-P_A_ and C_A_-F_A_) under the affected painful stimulus condition and all local and remote cortical networks of the unaffected side under the unaffected painful stimulus condition. Furthermore, the interconnections of C_A_-F_A_, C_U_-MT_U_, and C_U_-FP_U_ played a key role in CRS-R improvement. Therefore, the improvements in CRS-R and mGOS could be interpreted as an increase in the interconnections between the sensorimotor area and frontal area on the affected side as well as interconnections among the sensorimotor, middle temporal, and prefrontal areas on the unaffected side.

### Optimizing the Strategy of Multi-Target tDCS for Patients With Prolonged DOC

Transcranial Direct Current Stimulation has been used in the clinical treatment of and research on DOC for many years. However, most studies still use a single target to observe the efficacy of tDCS in DOC. This technique allows the therapeutic effect and possible mechanism of tDCS in the target area to be explained more clearly with fewer confounding factors. The observed transient improvements produced by tDCS in DOC were related to improvements in attention and working memory related to prefrontal cortical functioning (D’Esposito et al., 1998). Active tDCS applied to the OFC could improve the level of consciousness based on an improvement in residual connectivity among the prefrontal and motor areas (Naro et al., 2015). The stimulated left DLPFC area, which has a central integrative function, receives somatosensory and visual inputs from the parietal heteromodal association cortices regarding motion, vision, and tactile sensations, as well as spatial orientation, and projects to subcortical cholinergic and monoaminergic sources (Heekeren et al., 2006). A functional magnetic resonance imaging study indicated that tDCS over the left DLPFC increased functional connectivity in the “default mode” (intrinsic network) and bilateral frontal-parietal associative cortical networks (extrinsic networks) (Keeser et al., 2011), which are known to be involved in internal and external awareness, respectively (Vanhaudenhuyse et al., 2011). These studies have provided us with treatment references regarding different target areas and the corresponding research basis. However, clinical treatment is different from scientific research, and the former is more concerned about how to maximize the curative effect.

In this study, tDCS was applied simultaneously to the prefrontal area, left DLPFC, and bilateral FTPCs in patients with DOC to maximize the curative effect and optimize the treatment strategy. Considering the adverse reactions (i.e., epilepsy or spasticity) caused by long-term tDCS stimulation in one region, we designed an alternate left-right tDCS protocol, wherein the following targets were stimulated in sequence: the prefrontal area, left FTPC, right FTPC, and left DLPFC. As can be seen from the results, the median CRS-R score increased from 10 to 16 in the tDCS group. Simultaneously, the mGOS score increased by one point, indicating that the overall prognosis of the patients improved. These results were significantly better than those of the control group. Moreover, we did not apply hierarchical statistical analysis in patients with UWS and MCS, as did most of the previous studies. This could better reflect the therapeutic value of this tDCS treatment for DOC in general. Ultimately, it is obvious that the higher the CRS-R score, the better the prognosis of the patients. Our results also showed that the incidence of mGOS improvement in patients with MCS was five times higher than that in patients with UWS (Table 5).

### Multi-Session tDCS Treatment Is Necessary for Neural Plasticity and Functional Reorganization in Patients With Prolonged DOC

The rehabilitation process of prolonged DOC depends on neural plasticity and functional reorganization but with a little possibility of spontaneous recovery. Moreover, achieving behavioral improvements in a short time will be difficult in these patients. Many studies using limited sessions of tDCS illustrated transient improvements in consciousness in some patients with MCS but did not display the long-term effects of tDCS (Thibaut et al., 2014; Huang et al., 2017). Similarly, in this study, patients with prolonged DOC in the control group showed less improvement in the CRS-R scores (the group average was one point after the 2-month conventional treatment). However, several studies using multi-session tDCS (20 or 36 sessions) achieved better results in patients with MCS (Dimitri et al., 2017; Martens et al., 2018). Although no studies indicated the correlation between training time and structural brain changes, robust effects will be easier to produce if the training intervention is both intense and long-term (Caeyenberghs et al., 2018). Nevertheless, we cannot generally conclude that the more the tDCS sessions, the greater the improvement in the curative effect. In our pilot study, we had to discontinue the tDCS treatment because of the aggravation of spasticity caused by using more than two cycles of tDCS. Considering the risk and feasibility of the research, we, therefore, formulated a study plan including 80 sessions of tDCS. As can be seen from our research results, the patients in the tDCS group showed more significant improvements in both behavioral and electrophysiological evaluations than did those in the control group.

### Electrophysiological Effects of tDCS on Prolonged DOC

The multivariate logistic regression analysis showed that the incidence of mGOS improvement in the tDCS group was 2.42 (95% confidence interval: 1.03–5.68; *p* = 0.042) times higher than that in the control group (Table 5), indicating that multi-target and multi-session tDCS treatment improved the prognosis of patients. Compared to the control group, the tDCS group showed only significantly higher C_A_-P_A_ and C_A_-F_A_ under the affected painful stimulus condition, whereas all the local and remote C-ApEn indices of the unaffected hemisphere were significantly higher under the unaffected painful stimulus condition. This finding suggested that tDCS on the affected hemisphere mainly activated the local network, while that on the unaffected side more widely activated both the local and remote networks (Table 4). This further indicated that tDCS could better activate the local (i.e., on the affected hemisphere) and remote networks (i.e., on the unaffected hemisphere). Multivariate linear regression analysis revealed that C_A_-F_A_, C_U_-MT_U_, and C_U_-FP_U_ were the most relevant factors for CRS-R improvement. This suggested that the current study protocol improved the prognosis in prolonged DOC probably by improving the cortical connections between the M1 and frontal cortex of the affected hemisphere and the prefrontal-sensorimotor cortex and temporal-sensorimotor cortex associative cortical networks of the unaffected hemisphere.

Concurrently, we found that the change in the CRS-R score in the tDCS group was 1.278 points higher than that in the control group (*p* = 0.005) under the affected painful stimuli conditions, but no significant difference was observed in the changes in the CRS-R score between the two groups under the unaffected painful stimulus conditions (Tables 6, 7). To some extent, this means that tDCS treatment has a more obvious effect on the affected hemisphere. It also provides us future directions and references to optimize the treatment of DOC by using tDCS.

### Limitations

Although this study had a control group, and the sample size was more than 100, it was not a standard and rigorous RCT. Future studies should aim to further optimize the treatment plan and stimulation parameters of tDCS. The subscores of CRS-R were not listed due to missing data in early patients, otherwise, they would provide more information for the improvement of DOC. The follow-up in this study was also relatively simple and lacked any objective assessment indices (such as EEG). Moreover, EEG combined with other electrophysiological methods, such as event-related potentials which might provide more information on higher-order cortical information processing (e.g., P300), could further clarify the neural mechanism underlying the therapeutic effect of tDCS.

## Conclusion

The application of multi-session tDCS over the prefrontal area, left DLPFC, and bilateral FTPCs could improve the prognosis of patients with prolonged DOC. The recovery might be related to an improvement in the cortical connections between the M1 and the frontal cortex of the affected hemisphere and the prefrontal-parietal and temporo-parietal associative cortical networks of the unaffected hemisphere. Thus, tDCS may be an effective add-on treatment for patients with prolonged DOC.

## Data Availability Statement

The raw data supporting the conclusions of this article will be made available by the authors, without undue reservation.

## Ethics Statement

The studies involving human participants were reviewed and approved by the Ethics Committee of Xuanwu Hospital of Capital Medical University and the ethics committee of Wangjing Hospital of China Academy of Chinese Medicine Sciences. Written informed consent to participate in this study was provided by the participants’ legal guardian/next of kin.

## Author Contributions

XZ and BL made substantial contributions to the data analysis and drafting the manuscript. YL, GD, and JH treated the patients and acquired the data. DW designed the study and supervised and critically revised the manuscript. All authors approved the final manuscript.

## Conflict of Interest

The authors declare that the research was conducted in the absence of any commercial or financial relationships that could be construed as a potential conflict of interest.

## Publisher’s Note

All claims expressed in this article are solely those of the authors and do not necessarily represent those of their affiliated organizations, or those of the publisher, the editors and the reviewers. Any product that may be evaluated in this article, or claim that may be made by its manufacturer, is not guaranteed or endorsed by the publisher.
